# Combined treatment of TROP‑2 targeted CAR-T and vascular disruptor CBP enhances anti‑tumor activity in triple‑negative breast cancer

**DOI:** 10.1016/j.tranon.2026.102828

**Published:** 2026-05-29

**Authors:** Yizhu Chen, LiSheng Wang, Jian Jiang, YuFan Wei, Peng Jiao, JiaHu, WeiYuan Zhang, Jingjin Zhu, YiMing Wang, Xiru Li, Fengjun Xiao, Li Zhu

**Affiliations:** a Department of General surgery, The First Medical Center of Chinese PLA General Hospital, Beijing, China; b Key laboratory of Carcinogenesis and Translational Research (Ministry of Education), Anesthesiology, Peking University Cancer Hospital & Institute, Beijing, China; c Laboratory of Molecular Diagnosis and Regenerative Medicine, the Affiliated Hospital of Qingdao University, Qingdao, China; d Department of Rehabilitation, School of Nursing, Jilin University, Changchun, China; e School of Medicine, Nankai University, Tianjin, China; f Department of Thoracic Surgery, Qinghai Provincial People’s Hospital, Xining, China; g Department of Newborn Care Center, Senior Department of Pediatrics, The Seventh Medical Center of PLA General Hospital, Beijing, China; h Beijing Institute of Radiation Medicine, Beijing, China; i Department of Hepatobiliary Surgery, Union Hospital, Tongji Medical College, Huazhong University of Science and Technology, Wuhan, Hubei, China

**Keywords:** Chimeric antigen receptor T cells (CAR-T), Trophoblast cell surface antigen 2 (TROP-2), Breast cancer, Tumor cell immunotherapy, Vascular disruptor

## Abstract

**Objectives:**

This study aimed to investigate the synergistic potential of TROP-2-targeted CAR-T cells combined with the vascular disrupting agent PLG-g-mPEG/CA4/BLZ945 (CBP). We hypothesized that CBP would disrupt the tumor neovascular, thereby enhancing infiltration of the CAR-T cells.

**Methods:**

Analyzed TROP-2 expression in breast cancer using bioinformatics, qRT-PCR, and sequencing. Constructed and evaluated TROP-2 CAR-T cells in vitro. Finally, we prepared an animal model of breast cancer, and verified the anti-tumor therapeutic effect, safety evaluation and biologic mechanisms of TROP-2 CAR-T combined with CBP in vivo.

**Results:**

Bioinformatics analysis and in vitro experiments showed that TROP-2 was widely expressed in breast cancer cells. The successfully constructed TROP-2 CAR-T cells exhibited high cytotoxicity in vitro. The experimental results in mice showed that the combination therapy of CBP and TROP-2 CAR-T significantly enhanced the anti-tumor effect, and no significant decrease in survival rate or major organ damage was observed. Mechanism studies have shown that the anti-tumor effect of the combination therapy group may be related to immune cell infiltration, CAR-T cell infiltration, and upregulation of immune related factors.

**Conclusion:**

Our findings demonstrated the potential of TROP-2 as a viable target for CAR-T therapy in breast cancer. The combination of TROP-2 CAR-T cells with CBP not only enhances the therapeutic efficacy but also maintains a favorable safety profile, offering a promising new strategy for the treatment of breast cancer.

## Research highlights

• TROP-2 was a promising target for CAR-T cell therapy in breast cancer.
• The combination of TROP-2 CAR-T cells with PLG.CA4 Vascular Blocker markedly improved anti-tumor efficacy, without causing notable adverse effects.
• Substantial infiltration of T cells and macrophages into the peripheral blood and tumors was observed undergoing combination therapy.
• Upregulation in the expression of immune-related molecules was also observed, encompassing cytokines, apoptotic genes, and the infiltration of CAR-T cells.

## Graphical abstract

**Figure:**
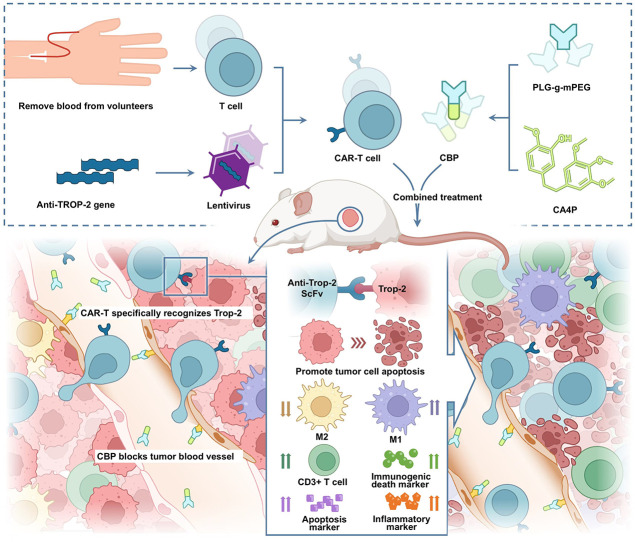

## Introduction

Breast cancer is the most common malignant tumor among women worldwide. For the HR+ and HER2+ subtypes, there are standard treatment regimens such as CDK4/6 inhibitors combined with endocrine therapy and new antibody-Drug Conjugate drugs (ADC drugs), which have significantly improved the prognosis [1]. Triple-negative breast cancer (TNBC), due to the lack of ER, PR, and HER2 expression, cannot benefit from endocrine and traditional HER2-targeted treatments, accounting for approximately 15% - 20% [2]. It is highly invasive and prone to recurrence and metastasis, with limited chemotherapy efficacy. Although immune checkpoint inhibitors and ADC have been used in some patients, the overall prognosis of metastatic TNBC remains poor [3]. Chimeric antigen receptor T-cell (CAR-T) therapy, as a new adoptive immunotherapy, provides a new treatment direction for TNBC [4].

CAR-T cell therapy has demonstrated considerable efficacy in treating hematological malignancies, but its effectiveness in treating solid tumors remains significantly limited [5,6]. This limitation is attributed primarily to the phenomenon of "T-cell exhaustion" observed in solid tumors, which leads to the functional impairment of T cells that infiltrate these tumors [7]. Moreover, the tumor microenvironment (TME) of solid tumors is notably intricate and comprises not only the tumor cells themselves but also tumor-associated stromal cells [[8], [9], [10], [11]]. The formation of new blood vessels is also critical within the TME [12]. These elements create a microenvironment that promotes tumor development and invasiveness while concurrently diminishing the anticancer efficacy of T cells through various mechanisms. Consequently, the infiltration of CAR-T cells into solid tumors is improved, and the characteristics of the TME are modified to mitigate T-cell exhaustion. Such strategies may facilitate CAR-T-cell therapy in overcoming the challenges associated with the treatment of solid tumors [13].

TROP-2, also known as TROP hoblast cell surface antigen 2, is overexpressed in a variety of solid tumors [[14], [15], [16]]. Particularly in breast cancer, the high expression of TROP-2 is closely associated with its key role in tumor growth, proliferation, and metastasis, as well as the poor prognosis of patients. The application of the TROP-2 target is becoming a hot topic for research and clinical attention. Sacituzumab govitecan, the first antibody‒drug conjugate (ADC) drug that targets TROP-2, has been approved for the treatment of advanced triple-negative breast cancer and has shown potential in HR+/HER2- breast cancer [17,18]. In addition to sacituzumab govitecan, several other TROP-2 ADC drugs are under clinical investigation [19,20]. Although they have shown preliminary efficacy in the treatment of breast cancer, the side effects of drugs are still serious issues that cannot be ignored. Researchers are exploring other therapeutic approaches targeting TROP-2 and combining them with other treatment regimens to improve therapeutic outcomes.

Combretastatin A-4 phosphate (CA4P) has the capacity to interact with microtubule proteins within the blood vessels of tumors, thereby compromising the structural integrity of the vasculature. This disruption ultimately leads to the occlusion of tumor blood vessels and the subsequent inhibition of tumor growth [[21], [22], [23]]. The utilization of vascular blockers in tumor therapy presents several limitations, including the reversible impact of CA4P on microtubule proteins and its rapid clearance from the plasma [[24], [25], [26]]. A novel polymer vascular blocker, designated PLG-g-mPEG/CA4/BLZ945 (CBP), employs polyglutamic acid grafted with polyethylene glycol (PLG-g-mPEG) as a delivery vehicle. The synthesis of CA4 and BLZ945 is achieved through Yamaguchi esterification, which covalently links these agents to the main chain of polyglutamic acid (PLG), resulting in the formation of CBP [27]. The drug loading capacities of CA4 and BLZ945 within CBP were quantified at 14.5 wt% and 6.5 wt%, respectively. This formulation addresses the current challenges associated with the application of CA4P by disrupting immature tumor blood vessels, thereby increasing the infiltration and viability of CAR-T cells.

This study initially involved the preparation of CAR-T cells targeting TROP-2 via gene transduction, followed by an assessment of the therapeutic efficacy of CBP in conjunction with TROP-2 CAR-T cells against breast tumors, which were utilized both in vitro and in vivo.

## Materials and methods

### Bioinformatics data processing and analysis

The bioinformatics platform available at http://gepia3.cancer-pku.cn was utilized to investigate and analyze the expression levels of TROP-2 across various tumor types. Specifically, we focused on the expression of TROP-2 in different subtypes of breast cancer and examined its correlation with prognosis and survival outcomes in breast cancer patients.

### Cell culture and extraction

This study utilized a variety of breast cancer cell lines, including MDA-MB-231, MDA-MB-453, HER2-positive cell line BT474, estrogen receptor-positive cell line MCF7, and luciferase-labeled MDA-MB-231-luc. Additionally, Raw 264.7 and HT1080 macrophage cell lines were used. These cells were all cultivated by our research group. MDA-MB-231-luc was initially purchased from NOBLEBIO and verified through luciferase activity detection. MDA-MB-231-luc, MDA-MB-231, MDA-MB-453, MCF-7, Raw 264.7, and HT1080 cells were all cultured in a regular DMEM medium (Gibco, Grand Island, USA) with 10% fetal bovine serum added. The BT474 cell line was cultured in specialized medium consisting of 1640 basic medium (Gibco, Grand Island, USA) supplemented with 15% special grade fetal bovine serum, 10 ml of insulin (400 units, 140X), sodium pyruvate (100X), and nonessential amino acids (100X) (Solarbio, Beijing, China). Stromal cells were isolated from tissue samples obtained from patients at the First Medical Center of the PLA General Hospital as part of a routine hospital procedure. The samples were washed with double-antibiotic physiological saline and subsequently minced. A standard digestion solution comprising 0.2% type I collagenase (Gibco, Grand Island, USA), 0.25% trypsin (Gibco, Grand Island, USA), and PBS (Biosharp, Beijing, China) was added to the tissue samples, which were then placed on a shaking platform at 37 °C for 6 h at 120 rpm. Following digestion, the cells were resuspended in human mesenchymal stem cell (MSC) culture medium supplemented with 1% penicillin‒streptomycin solution (Solarbio, Beijing, China). All the cell lines were incubated in a 5% CO2 atmosphere at 37 °C for further cultivation. In accordance with the Declaration of Helsinki (2013 revision), this study received approval from the Ethics Committee of Chinese PLA General Hospital (No. S2024– 675– 02), and written informed consent was obtained from all individual participants.

### Transcriptomic sequencing

Tumor-associated fibroblasts exhibiting optimal growth conditions were harvested, treated with TRIzol reagent, and then transported on dry ice to Annouda for sequencing. An in-depth analysis of differential gene expression was conducted via the fragments per kilobase per million mapped fragments (FPKM) method. The study implemented standard criteria for differential gene expression screening, defined by a | log2-fold change | ≥ 1 and q < 0.05.

### Immunohistochemical staining of TROP-2

Breast cancer and paracancerous tissue samples were collected from the First Medical Center of PLA General Hospital. The samples were fixed in formalin, embedded in paraffin, sectioned and stained with anti-TROP-2 (ABclonal, Wuhan, China). Images were obtained via an optical microscope (Leica, Wetzlar, Germany). Quantitative analysis was conducted via ImageJ software.

### Human T-cell isolation and modification

We developed this vector by integrating the modified TROP-2 specific single-chain variable fragment (scFv) into the pLVX-EF1α-Con background vector. Eventually, we prepared the pLVX-EF1α-TROP-2 CAR expression lentiviral plasmid, where the single-chain variable fragment (scFv) sequence of the chimeric antigen receptor (CAR) vector originated from an antibody drug currently in patent application. The construction of this vector was carried out by Sanly-Health Cell Technology Inc., Beijing 102,615, China. Next, the lentiviral vector (16 mg), the psPAX2 packaging plasmid (12 mg), and the pMD2.G envelope plasmid (4 mg) was co-transfected into HEK293T cells using the polyethyleneimine (PEI) transfection method. The supernatant was collected, then concentrated using PEG reagents, and the virus titer was detected using HT1080 cells. The virus titer could reach 1 × 10^8^, and the concentrated virus solution was stored at −80 °C until use. Peripheral blood samples were obtained from healthy volunteers and isolated after obtaining informed consent. Monocytes were separated by differential centrifugation, while undisturbed T cells were isolated using the Dynabeads™ Undisturbed™ Human T Cells Kit (Invitrogen, California, USA). The T cells obtained by negative selection were resuspended in T cell culture medium (Tongli Haiyuan Biotech, Beijing, China), and the cell density was adjusted to 5 × 10^7^/ml cells. CD3/CD28 magnetic beads (Tongli Haiyuan Biotech, Beijing, China) and 100 IU/ml IL2 (Tongli Haiyuan Biotech, Beijing, China) were added for a 30-minute incubation at room temperature. The cell density was adjusted for plating, and the cells were changed every two days for counting. After three days of culture, the activated T cells were collected, the supernatant was removed by centrifugation, counted, and resuspended in the immunocyte-specific culture medium, along with 100 IU/ml IL2 and 5 ug/ml Polybrene. The TROP-2 CAR lentiviral vector was used for transduction at an MOI of 40, and transfection was performed for 12 h. Afterward, the medium was changed for continued culture. On the 7th day after infection, the expression of the CAR molecule was detected. The car-t cells were stained using Biotinylated Human Trop-2/TACSTD2 Protein (Acro Biosystem, Newark, USA) and APC-streptavidin (BD Biosciences, San Jose, USA), and the proportion of positive cells expressing Trop-2 scFv was determined using a flow cytometer and the data were analyzed using FlowJo software.

### CAR-T-cell killing experiment in vitro

The T cells were cultured and transfected at an appropriate infection multiplicity of infection (MOI). Meanwhile, 231-LUC cells, MDA-MB-231 cells, MDA-MB-453 cells, BT474 cells, and MCF7 cells were inoculated in 96-well plates at the same density to ensure experimental reproducibility. The corresponding effector cells (CAR-T cells) were introduced into the corresponding wells at a 1:1, 5:1, and 10:1 effector cell: target cell (E:T) ratio with 231-LUC cells, and at a 1:10 ratio with MDA-MB-231, MDA-MB-453, BT474, and MCF7 cells. They were mixed and co-cultured respectively. Three replicates were set for each cell line and each E:T ratio condition, and negative control wells and positive control wells were also set. The blank control well was included. After 24 h of co-culture, the cells were divided into two groups for detection: one group prepared the lysis buffer and luciferase substrate (Lablead, Beijing, China) and then analyzed to obtain fluorescence measurement values, so as to evaluate the cytotoxicity of CAR-T cells against 231-LUC cells under different E:T ratios; the other group used the LDH cytotoxicity assay kit, following the kit instructions, collected the supernatants from each well, added LDH detection reagent, incubated in the dark for a certain period of time, and measured the absorbance of each well using an enzyme reader at 450 nm wavelength. The cytotoxicity (%) of MDA-MB-231, MDA-MB-453, BT474, and MCF7 breast cancer cell lines was calculated using the formula: Cytotoxicity (%) = (OD treatment group - OD blank control group - OD negative control group) / (OD positive control group - OD negative control group) × 100%.

### Animals

In this study, 5-week-old SPF-grade NTG mice were purchased from Sipeifu (Beijing) Biotechnology Co., Ltd. The average weight of these mice was 19 g. The mice were raised in the standard animal room of the Academy of Military Medical Sciences. The ambient temperature of the standard animal room was 23 °C, the humidity was 55%, and the light cycle was 12 h/12 hours. Five mice were raised in each cage. Each cage was equipped with an independent air purification system. The feed and water used were sterilized and replenished in time. They were raised adaptively for approximately one week before subsequent experimental operations. After the animal experiments were completed, the mice were euthanized via the cervical dislocation method. The animal welfare and experimental procedures of this study were carried out in accordance with the guidelines for laboratory animals, handling norms, and animal ethics, as well as the ethical standards of our institute (Animal Ethics Approval Number: IACUC-DWZX-2023– 503).

### Construction of a tumor-bearing model

Six-week-old NTG mice weighing approximately 18–20 g were utilized. MDA-MB-231 cells were combined with a matrix gel (Corning, New York State, USA) and PBS at a 1:1 ratio, resuspended to a concentration of 7 × 10^6^ cells per 200 µL, and subsequently inoculated subcutaneously in the axillary region of the mice. Approximately seven days post-inoculation, when the tumor volume reached 100 mm³, the mice were randomly assigned to four treatment groups (n = 6). This time point is Day 0, and the specific treatment groups and methods are detailed in Supplementary File 3. After receiving CBP (20 mg/kg body weight) treatment, CAR-T cell therapy (5 × 10⁶ cells per mouse, 200 microliters) was immediately injected. This study aimed to compare tumor growth kinetics across various treatment groups by monitoring tumor volume (calculated as V = (length × width²)/2). Body weight was also monitored during the observation period. Additionally, Kaplan‒Meier survival curves were generated for each group. On the 23rd day following treatment, the mice were euthanized, and tumor specimens, along with multiple organ tissues and blood samples, were collected for analysis of immune-related indicators.

### Flow cytometry

Peripheral blood was extracted from the mice and subsequently divided into two groups for labeling with specific antibodies following anticoagulation, including CD3-BV605 (BioLegend, California, USA), and F4/80-FITC (BioLegend, California, USA). The samples were incubated at 4 °C in the dark for 30 min, followed by red blood cell lysis at room temperature for 10 min. The samples were then centrifuged and resuspended for analysis.

### Routine peripheral blood analysis

Venous plexus blood was collected from the mouse eye socket and anticoagulated, and 20 µL was used for blood count analysis via an automated machine (Sysmex, Kobe, Japan). The key indicators used to assess biological safety included HBG, WBC, RBC, and PLT.

### Hematoxylin and eosin (H&E) staining and multicolor immunohistochemistry

Tissue samples obtained from the mice were fixed in a 4% formalin solution and subsequently embedded in paraffin. The embedded tissues were then sectioned into continuous slices with a thickness of 4 μm. Sections derived from various organs, including the heart, liver, spleen, and lungs of mice, were subjected to H&E staining to assess tissue morphology and safety profiles. For multicolor immunohistochemistry, the Opal™ 4-Color Anti-Rabbit Manual IHC Kit (Akoya, Massachusetts, USA) was used to concurrently detect the expression levels of multiple immune cell markers within murine tumor tissues.

### Fluorescent quantitative nucleic acid amplification detection

Fluorescent quantitative nucleic acid amplification detection was conducted utilizing specifically designed primers (Supplementary file 3). Cellular RNA was extracted with TRIzol reagent (Invitrogen, California, USA), and DEPC-treated water was utilized to maintain RNA integrity. The concentration of RNA was determined via a Nano300 specTROPhotometer (Allsheng, Hangzhou, China). Reverse transcription into complementary DNA (cDNA) was performed with TransScript® One-Step gDNA Removal (TransGen Biotech, Beijing, China) and cDNA Synthesis SuperMix (TransGen Biotech, Beijing, China). Quantitative PCR (qPCR) was performed via PerfectStart Green qPCR SuperMix.

### Statistical analysis

All statistical analyses were performed using GraphPad Prism 9.0 software. Quantitative data were presented as mean ± standard error of the mean (SEM). The Shapiro–Wilk test was applied to evaluate the normality of data distribution, and Levene’s test was used to assess homogeneity of variance. For data with normal distribution and homogeneous variance, unpaired *t*-test was used for comparisons between two groups, and one‑way ANOVA followed by Tukey’s multiple comparisons test was adopted for multi-group analysis. Data that did not conform to a normal distribution were analyzed using non-parametric methods. A P value < 0.05 was defined as statistically significant.

## Results

### Expression analysis of TROP-2 in breast cancer

**Fig. 1:**
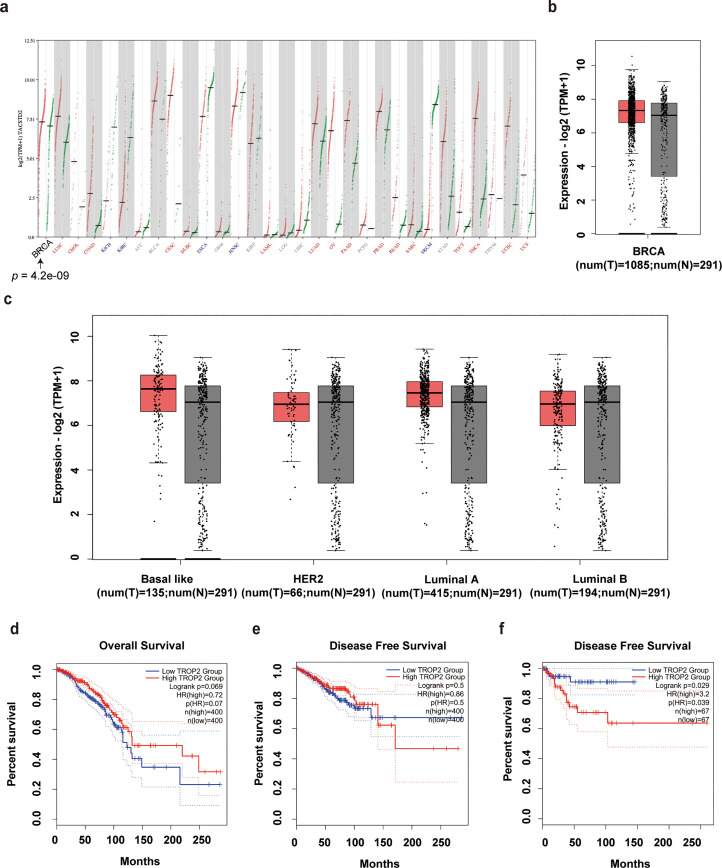
Bioinformatics analysis of TROP-2 in breast cancer. (a). TROP-2 pan-cancer analysis. (b). Expression of TROP-2 in breast cancer. (c). The expression of TROP-2 in different molecular types of breast cancer. (d). Overall survival of breast cancer with different TROP-2 expression. (e). Disease free survival of breast cancer with different TROP-2 expression. (f). Disease free survival of triple negative breast cancer with different TROP-2 expression.

This investigation employed an online bioinformatics platform to assess the expression of the TROP-2 molecule across various cancer types. The findings indicated that TROP-2 was significantly overexpressed in multiple tumors, including cervical, thyroid, urothelial, lung, liver, pancreatic, and ovarian cancers (Fig. 1a). However, in breast cancer, while there was an increase in TROP-2 expression, it was not statistically significant (Fig. 1b). To further delineate the expression levels of TROP-2 across different molecular subtypes of breast cancer, we observed an increase in TROP-2 expression across these subtypes (Fig. 1c and d). We subsequently examined the correlation between TROP-2 expression and the prognosis and survival outcomes of breast cancer patients. The analysis revealed that TROP-2 expression was not significantly associated with overall survival (OS) or disease-free survival (DFS) in the general breast cancer cohort. However, in the context of TNBC, elevated TROP-2 expression was linked to a reduction in DFS (Fig. 1e and f). These findings suggest that TROP-2 may represent a valuable therapeutic target in the treatment of TNBC, warranting further investigation.

**Fig. 2:**
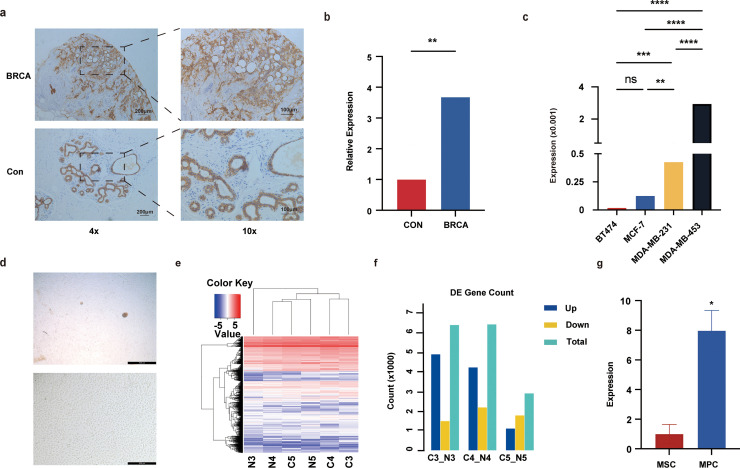
TROP-2 expression in breast cancer. (a). Immunohistochemical detection of TROP-2 expression in breast tissue (4x, 10x). (b). Quantitative analysis of TROP-2 expression levels in breast tissue (***, *p* < 0.001). (c). The expression of TROP-2 in various breast cancer cell lines was detected by qPCR. (d). Morphological characteristics of mesenchymal precursor cells (MPCs) (4x, 10x). (e). Heatmap of gene expression in three different MPCs. (f). Bar chart of differentially expressed genes among the three MPC sample groups. (g). The expression level of TROP-2 in stromal cells (*, *p* < 0.05).

Additionally, we analyzed patient samples obtained from the breast surgery department of the General Hospital of the Chinese People's Liberation Army (n = 11). The results indicated that TROP-2 was predominantly expressed in the ductal epithelium of normal breast tissue, albeit at relatively low levels. In contrast, TROP-2 expression in breast cancer tissue was heterogeneous, with notably high expression levels (Fig. 2a). Quantitative analysis via immunohistochemistry revealed that the TROP-2 content in breast cancer tissue was approximately double that in normal breast tissue (Fig.2b), suggesting that TROP-2 may serve as a specific target for breast cancer. We also conducted quantitative PCR (qPCR) to evaluate TROP-2 expression in established breast cancer cell lines, including MDA-MB-231, MDA-MB-453, MCF-7, and BT474. The results indicated that TROP-2 was expressed in all the examined cell lines, with the most pronounced expression observed in the TNBC cell lines (Fig. 2c).

### Analysis of TROP-2 expression in the tumor microenvironment of patients with breast cancer

The tumor microenvironment is integral to the efficacy of cancer treatment. This study aimed to further elucidate the expression of TROP-2 within this microenvironment. We isolated mesenchymal precursor cells (MPCs) from tumor samples obtained from patients and observed that these cells adhered to the culture vessel. Upon attachment, the cells exhibited a spindle or elongated spindle morphology, characterized by a larger cell size and a vortex-like appearance. High-magnification microscopy revealed an oval-shaped nucleus centrally located within the cell, featuring a prominent nucleolus (Fig. 2d). Research has shown that tumor-associated fibroblasts possess multiple types of differentiation potential and specific marker expression. Following validation, we employed transcriptome sequencing technology to conduct a differential analysis of patient samples, quantitatively assessing the resulting gene sequencing data (Fig. 2e and f). Differentially expressed genes were subsequently selected for qPCR experiments to confirm the reliability of the transcriptome sequencing findings. The sequencing results indicated that TROP-2 was also highly expressed in MPCs, suggesting that TROP-2 may serve as a target for the selective elimination of certain stromal cells within the TME. Further qPCR analysis of TROP-2 expression in MPCs revealed significant upregulation, with expression levels approximately five times greater than those observed in normal breast tissue stromal mesenchymal stem cells (n = 4, Fig. 2g). Therefore, we hypothesize that the CAR-T cell therapy can effectively target tumor cells and simultaneously eliminate the tumor stromal cells that provide protective effects, thereby enhancing the treatment strategies for breast cancer. However, further conclusions require more in-depth experimental verification.

### Construction and evaluation of TROP-2 CAR-T cells

**Fig. 3:**
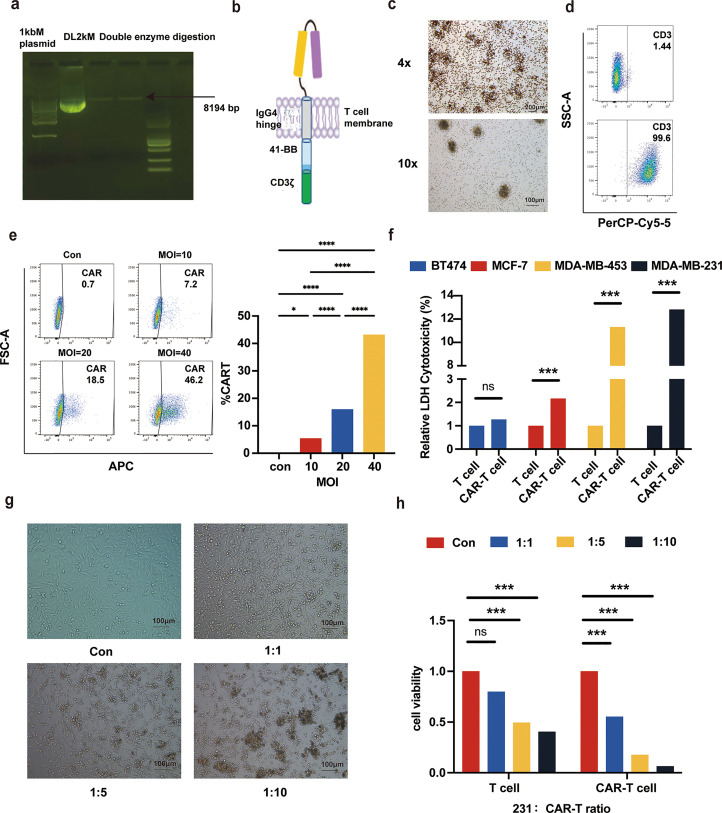
Construction and detection of TROP-2 CAR-T cells. (a). CAR plasmid electrophoresis detection. (b). Schematic diagram of CAR molecules on the surface of T cells. (c). Observation of T cells under a microscope (4x, 10x). (d). Detection of the CD3 positivity rate in T cells. (e). CAR-T-cell yield under different multiplicities of infection. (f). Relative LDH cytotoxicity of T cells and CAR-T cells therapy in different breast cancer cells. (g) Killing effects of CAR-T cells and tumor cells at different effector-to-target ratios (10x). (h). Quantitative analysis of the cytotoxicity of CAR-T cells and tumor cells at different target-to-effect ratios (10x) (**, *p* < 0.001). MOI: multiplicity of infection.

This investigation employed the empty vector pLVX-EF1α-Con as the foundation plasmid, alongside the second-generation CAR molecule pLVX-EF1α-Con, which encompasses the IgG4 hinge region, the CD3ξ motif, and the costimulatory signal region of the 4–1BB intracellular domain, to engineer TROP-2 CAR-T cells. The TROP-2 antibody ScFv was successfully integrated into the pLVX-EF1α-Con sequence, resulting in the generation of a lentiviral plasmid designated pLVX-EF1α-TROP-2-CAR. Following sequencing verification, the viral plasmid was effectively packaged (Fig. 3a). A schematic representation of the structure of the CAR molecule on the surface of T cells was subsequently generated on the basis of the pLVX-EF1α-TROP-2 CAR molecule structure (Fig. 3b). After viral encapsulation, the HT1080 cells were infected with the lentivirus via the following formula: infection titer = -ln (percentage of uninfected cells) × number of cells per well × dilution factor. The resulting viral titer exceeded 1 × 10^8^ (Supplementary file 1. Fig. S1), enabling the preparation of multiple batches of high-titer CAR lentivirus for CAR-T-cell generation. Furthermore, peripheral blood T lymphocytes were extracted from healthy volunteers and cultured for validation purposes. T cells exhibited a small, circular or ellipsoidal morphology characterized by suspended growth, clustered proliferation, and logarithmic growth, with a relatively rapid proliferation rate (Fig. 3c). On the fourth day, flow cytometry was used to assess the purity of the CD3-positive cells, and the percentage of positive cells exceeded 99.6% (9387/9421) (Fig. 3d). The high purity of the cells made them suitable for CAR-T-cell preparation for subsequent experiments. Following 24 h of T-cell activation, anti-TROP-2 CAR lentivirus was introduced at multiplicities of infection (MOIs) of 10, 20, and 40. After seven days of culture, flow cytometry was conducted to evaluate the positive expression rates of the CAR-T cells in each group, yielding rates of 7.2% (612/8497), 18.5% (1542/8339), and 46.2% (3984/8623) (Fig. 3e). The appropriate infection ratio (MOI) required for the subsequent experiments was determined through the gradient infection method. Finally, an MOI value of 40 was selected for further research. Firstly, through LDH cytotoxicity detection, it was found that the anti-TROP-2 CAR-T cells had a significant killing effect on four breast cancer cell lines (MDA-MB-231, MDA-MB-453, BT474, and MCF7), and the differences between groups were statistically significant (p < 0.05) (Fig. 3f). Among them, the killing effect on MDA-MB-231 cells was the most significant. On this basis, we further conducted luc detection on MDA-MB-231-LUC cells, using untransfected T cells as the control group. The results showed that the killing effect of the anti-TROP-2 CAR-T cells on 231-LUC cells was significantly better than that of the untransfected T cells. When the ratio of effector cells to target cells was 1:10, the killing rate of CAR-T cells could reach up to 94%. At the same time, microscopic observation showed that the anti-TROP-2 CAR-T cells would gather around the tumor cells. When attacked by CAR-T cells, the tumor cells showed abnormal signs, detaching from the attached state and aggregating with the CAR-T cells (Fig. 3g). The quantitative analysis results of fluorescence absorption were also consistent with the above observations and detection results, and the differences between groups were statistically significant (p < 0.001) (Fig. 3h).

### Antitumor effects of CBP combined with CAR-T cells in vivo

**Fig. 4:**
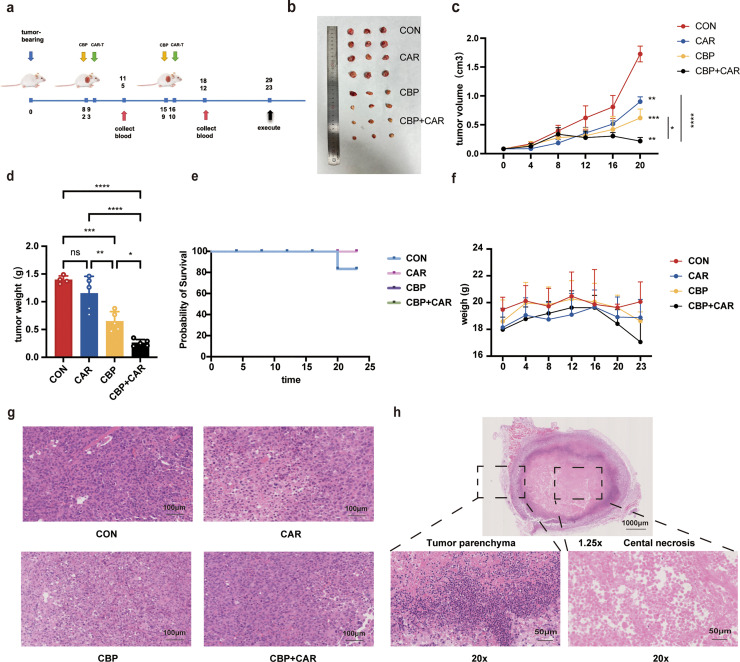
Effect of CBP combined with CAR-T cells in mice. (a). Experimental design timeline. (b). Tumor images of the mice in each experimental group after 23 days. (c). Changes in tumor volume growth in each group of mice over 23 days. (d). The tumor weight of each group of mice at 23 days (*, *p* < 0.05; **, *p* < 0.01; ***, *p* < 0.001; ****, *p* < 0.0001). (e). Twenty-three-day survival of the mice in each experimental group. (f). 23-day weight line chart for each group of mice. (g). H&E staining of tumors from each group of mice (10x). (h). H&E staining of central tumor necrosis in CBP combined with CAR-T-cell-treated mice (1.25x, 20x).

This investigation employed the vascular disruptor CBP as an adjunct to CAR-T-cell therapy. Tumor implantation was successfully conducted on a cohort of 20 mice (n = 6), resulting in consistent tumor sizes across subjects. Tumor dimensions were assessed every three days post-administration, and tumor volume was calculated via a specified formula (Fig. 4a). The results revealed statistically significant differences in tumor volume among the experimental groups, with the CBP and CAR-T combination groups demonstrating a marked reduction in tumor size (Fig. 4b and c). Following a 23-day observation period, the mice were euthanized, and the tumor weights were recorded at the conclusion of the study. Notably, the CBP and CAR-T-cell groups presented a tumor weight that constituted only 18% (0.26 g/1.43 g) of that of the PBS control group (Fig. 4d). Throughout the 23-day duration, only one mouse from the PBS group died, whereas no fatalities were reported in the other groups, suggesting that the treatment regimen was effective with minimal adverse effects (Fig. 4e). Tumor size was monitored concurrently with body weight measurements via an electronic balance, revealing no significant differences in body weight across the groups (Fig. 4f). However, a slight reduction in body weight was noted in the CBP and CAR-T-cell groups, which may be attributed to the stress induced by the CBP treatment. Subsequent histological analysis via hematoxylin and eosin (H&E) staining of tumor samples was conducted to evaluate their pathological characteristics. At low magnification, the tumor cells displayed diffuse sheet-like proliferation with indistinct boundaries. At higher magnification, the cells exhibited moderate to severe atypia, with visible pathological mitotic figures (Fig. 4g). Examination of the tumor tissue posttreatment revealed that the overall specimen section presented a gray‒white, solid, and tough texture, with poorly defined margins relative to adjacent tissues. Necrotic material, characterized by a gray‒yellow, brittle texture, was occasionally observed at the center of the tumor. At low magnification, extensive necrosis with distinct boundaries between the tumor center and surrounding tissues was evident, whereas high magnification revealed large areas of powder-stained amorphous material, indicative of a posttreatment response (Fig. 4h).

### Safety evaluation

**Fig. 5:**
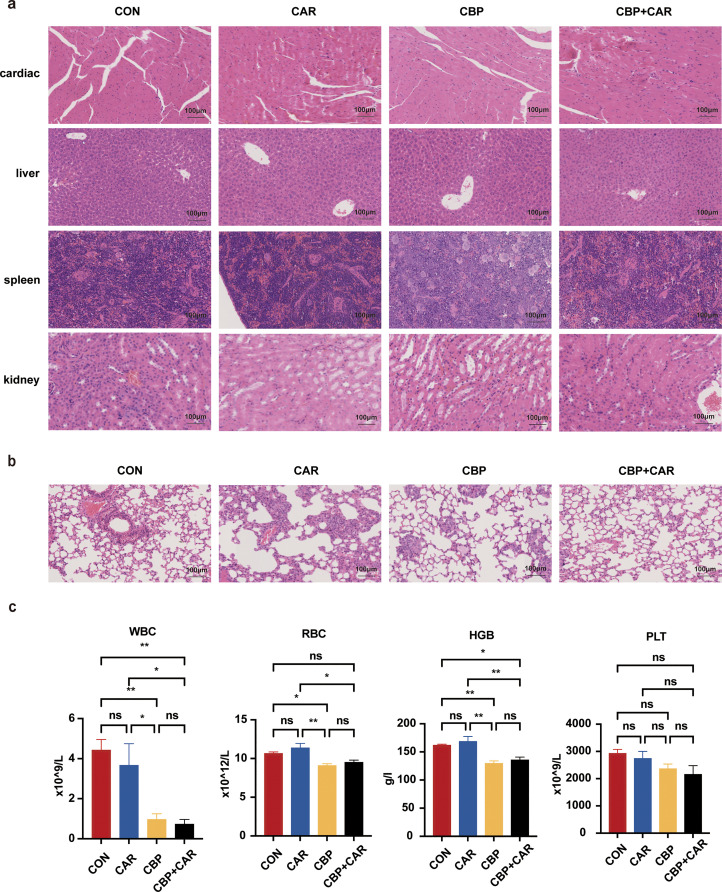
Safety evaluation of CBP combined with CAR-T cells in mice. (a). H&E staining of multiple organs in each group of mice (10x). (b). H&E staining of lung tissue from each group of mice (10x). (c). The results of routine blood examination in each group of mice (*, *p* < 0.05. **, *p* < 0.01).

Histological analysis via H&E staining revealed that the combination therapy did not have significant toxic effects on any of the examined organs. Microscopic evaluation of H&E-stained tissues revealed that all the tissue structures remained intact and that the cellular morphology appeared healthy (Fig. 5a). However, the immunocompromised state of the mice resulted in the loss of the splenic architecture, characterized by the absence of a discernible white pulp area and splenic corpuscles. Notably, atypical cells were observed in the lung tissues across all experimental groups, suggesting the occurrence of lung metastasis. Among the treatment groups, the morphology of the lung tissue in the group receiving CBP in conjunction with CAR-T-cell therapy was notably more preserved, and the incidence of lung nodules was reduced, indicating that the therapeutic efficacy of the combination treatment was superior to that of the other treatments (Fig. 5b). Additionally, this study further investigated the safety of the drug through peripheral blood routine tests, which revealed a reduction in hemoglobin (HBG) (*p* *=* 0.0297), red blood cells (RBCs) (*p* *=* 0.0021), and white blood cells (WBCs) (*p* *=* 0.0014) in the CBP-treated group (Fig. 5c). These findings suggest that CBP may induce certain adverse effects, such as anemia.

### Mechanism exploration

**Fig. 6:**
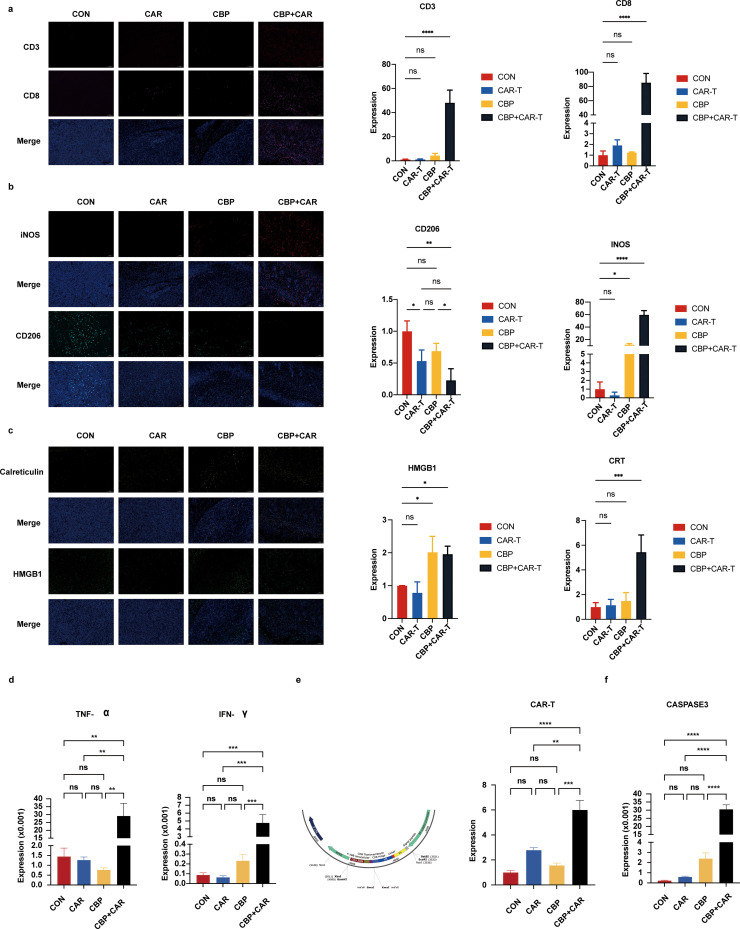
CBP combined with CAR-T-cell therapy for tumor immune cell expression in mice. (a). Multicolor immune detection of T-cell expression in the tumors of the mice in each group (10x). (*, *p* < 0.05; **, *p* < 0.01; ***, *p* < 0.001). (b). Multicolor immune detection of Macrophage expression in the tumors of the mice in each group (10x).(*, *p* < 0.05;**, *p* < 0.01; ***, *p* < 0.001). (c). Multicolor immune detection of HMGB1 and Calreticulin expression in the tumors of the mice in each group (10x).(*, *p* < 0.05;**, *p* < 0.01; ***, *p* < 0.001).. (d). Infiltration of immune factors within tumor tissues. (e). Detection of the degree of infiltration of CAR-T cells into tumor tissue on the basis of the WPRE structure. (f). Expression of apoptosis-related genes in tumor tissue (**, *p* < 0.01; ***, *p* < 0.001; ****, *p* < 0.0001).

Peripheral blood was extracted from murine subjects to assess immune cell populations. Notably, the group receiving combined treatment with CBP and CAR-T-cell therapy exhibited a significant increase in CD3^+^ T cells and increased expression of macrophages. It was tentatively proposed that the CBP combination therapy may facilitate antitumor effects through the promotion of immune cell aggregation (Supplementary file 2. Fig. S2). Further analysis was conducted to evaluate the infiltration of immune cells within the tumor microenvironment. Multicolor immunofluorescence analysis revealed a marked increase in the infiltration of T cells, particularly CD8^+^ T cells, within the CBP and CAR-T-cell treatment cohorts (Fig. 6a). Concurrently, there was an increase in M1 macrophage expression alongside a decrease in M2 macrophage expression (Fig. 6b). Additionally, we assessed markers indicative of immunogenic cell death and detected elevated levels of tumor high mobility group box 1 (HMGB1) and calretinin (CRT) (Fig. 6c). An analysis of inflammatory factor secretion within the tumors revealed increases in tumor necrosis factor alpha (TNF-α) (*p* < 0.001) and interferon gamma (IFN-γ) (*p* < 0.001) in the CBP combination therapy group (Fig. 6d). To further investigate the infiltration of CAR-T cells within tumors, we employed an indirect approach utilizing gene sequences of WPRE cis-acting elements, allowing for the detection of CAR-T-cell expression through qPCR experiments. The results revealed significant retention of CAR-T cells in the tumors of the CBP combination treatment group, which was approximately six times greater than that observed in the PBS treatment group (Fig. 6e). Moreover, an examination of apoptotic gene expression within the tumors revealed an increase in apoptotic gene activity in the combination therapy group (Fig. 6f), suggesting that the combined treatment may enhance the apoptotic processes in tumor cells.

## Discussion

TROP-2 was an important and specific therapeutic target for breast cancer. In this study, TROP-2 CAR-T cells were prepared and their anti-tumor effect on TNBC cells in vitro were explored. This study found that the combination of TROP-2 CAR-T cells and the vascular destroyer CBP could effectively inhibit tumor growth and metastasis in vivo.

TROP-2 was often overexpressed in various malignant tumors and was associated with poor prognostic outcomes [28]. Clinical studies on TROP-2 mainly focused on antibody-drug conjugates (ADCs) [17,18,29,30]. Among them, the clinical efficacy of satezumab govertecan in metastatic TNBC was remarkable and has been approved by the FDA [31]. Our results indicated that TROP-2 was significantly overexpressed in TNBC, and the expression of TROP-2 was associated with the DFS of TNBC patients. The samples of breast cancer patients in our hospital were analyzed, and the results showed that the expression level of TROP-2 in their tumor tissues was higher than that in normal tissues. Studies have found that although TROP-2 was overexpressed in various solid tumors, its expression levels showed significant heterogeneity among different tumor types and even different subtypes of the same tumor. Due to the limited number of patient specimen tissues in this study, subgroup analysis cannot be conducted for the time being. In the future, patient specimens could be further collected to deeply explore the expression of TROP-2 and its relationship with prognosis. At present, a variety of novel TROP-2 targeting strategies, including ADC and CAR-T, are underway, and the research scope has expanded from breast cancer to multiple tumor types such as non-small cell lung cancer, urothelial carcinoma and gastric cancer, etc.  [32]. Clinical data of sacituzumab govitecan showed that it still demonstrated certain clinical benefits even in patients with different levels of TROP-2 expression, which may be partly attributed to the “bystander effect” of the ADC [33]. However, CAR-T cell therapy does not have this bystander effect. Its anti-tumor activity strictly depends on antigen recognition. Therefore, the heterogeneity of TROP-2 expression may have a more severe impact on CAR-T therapy than on ADC [34]. It is worth noting that TROP-2 was also baseline expressed in some normal tissues, such as epithelial tissues of the lungs, kidneys, skin, etc. , suggesting that it may have off-target toxicity [[35], [36], [37]]. Research found that another member of the TROP-2 family, EpCAM (TROP-1/TACSTD1), had a high degree of structural homology with TROP-2 and might compensate for some of the functions of TROP-2, providing a certain level of safety for TROP-2-targeted therapy. However, this compensatory mechanism may also become a source of tumor drug resistance, leading to antigen escape and treatment failure [38]. In our study, we did not explore the dynamic changes in TROP-2 expression in tumor cells or the activation of alternative escape mechanisms after TROP-2 CAR-T cell therapy. This was an important topic that required systematic research in the future. Although TROP-2 was a clinically validated and valuable therapeutic target, its inherent limitations as a CAR-T cell therapy target, such as non-necessity of the target, heterogeneity of expression, and normal tissue expression, required that when promoting TROP-2 CAR-T therapy, we need to develop patient screening strategies and plans to deal with antigen escape in tandem.

The mechanism of traditional ADC drugs was limited by factors such as the immunosuppressive TME, poor internal tumor permeability, drug resistance, and non-targeted toxicity, which could impair long-term therapeutic effects and patient survival [39]. The synergistic strategy of TROP-2 CAR-T and CBP could achieve dual-targeted intervention. Through the antigen-specific cytotoxic effect of CAR-T cells and the killing mechanism mediated by targeted immunity, malignant cells expressing TROP-2 could be specifically identified and eliminated. CBP was used to destroy abnormal tumor neovascularization, alleviate hypoxia within the tumor, reshape the immunosuppressive TME, reduce the infiltration of myeloid-derived suppressor cells and regulatory T cells, and significantly enhance the infiltration, persistence and effector function of CAR-T cells in solid tumors [40]. Meanwhile, CAR-T cells could undergo clonal expansion in situ after antigen recognition, establish long-term immune surveillance, and mediate sustained anti-tumor activity, which was crucial for eliminating minimal residual lesions, preventing tumor recurrence and distant metastasis [41]. In addition, the combined strategy could expand the applicable patient group, including breast cancer patients who were resistant or drug-resistant to ADCs, reduce systemic toxicity through localized treatment effects, and provide a more durable and personalized immunotherapy regimen. Our research demonstrated the anti-tumor effect of the combined application of CBP and CAR-T cell therapy in vivo. However, a reduction in the number of white blood cells, hemoglobin and red blood cells was found in CBP and the combination treatment group, suggesting that CBP might have an inhibitory effect on bone marrow hematopoietic function. However, this hypothesis required bone marrow smear or hematopoietic stem cell analysis for further verification. CA4P, as the parent compound of CBP, has also been reported to have adverse events such as bone marrow suppression in clinical studies [42]. This blood toxicity might have a superimposed effect with CAR-T cell therapy for patients who are already in an immunosuppressed state, increasing the risk of infection and bleeding [43]. Therefore, the further optimization of CBP should focus on improving the surface modification of nanoparticles, enhancing the specific accumulation of drugs at tumor sites, optimizing the administration regimen, and determining the optimal timing combination of CBP and CAR-T cell infusion, etc. Comprehensive hematological monitoring and bone marrow histopathological assessment should be included in subsequent studies to more accurately characterize the hematological toxicity mechanism and reversibility of CBP.

We conducted a preliminary study on the anti-tumor mechanism by detecting the changes in the proportion of immune cells in the peripheral blood of mice. The results showed that the combination treatment group could effectively activate immune cells, and the infiltration of immune cells in the tumor was increased, which was significantly correlated with the tumor killing activity. By examining DAMPs, CRT and HMGB1 release on the surface of exposed cells, it was found that the combination therapy may be able to enhance the immune response by inducing ICD. CBP contributed to the survival and infiltration of CAR-T cells within the tumor, which might solve the challenge of CAR-T cell therapy in solid tumors by further disrupting the suppressive microenvironment of solid tumors. In addition, the immune response elicited by drugs in tumors was often associated with the release of immune factors [44]. Our results also showed that the combined therapy could promote the release of tumor cytokines, such as TNF-α and IFN-γ. TNF-α is an inflammatory cytokine that plays a key role in regulating immune responses, mediating tissue damage caused by T cells, and influencing chronic inflammatory responses as well as tumor development and progression. IFN-γ, the only type II interferon, is involved in cellular antiviral, antitumor, and immunomodulatory functions. The results of these experiments suggested that the antitumor effect of combination therapy may be related to the activation of immune factors [45].

The absence of a functional adaptive immune system in the NTG mice used in this study may mask the vascular remodeling induced by CBP and the potential interaction between CAR-T cells and host-derived immune cells, which may have a certain impact on our evaluation of the true efficacy and safety of combination therapy [46]. However, NTG mice still retain intact innate immune components, including macrophages, natural killer cells, dendritic cells and granulocytes, which enabled us to observe the innate immune activation induced by CBP and CAR-T, such as M1 macrophage polarization, elevated proinflammatory cytokines and increased immune cell death markers [47]. The CD8+T cell infiltration detected in the tumor tissue also proved that CBP promoted the infiltration and retention of CAR-T cells, which was critical for the synergistic antitumor effect. A sample size of only 5 mice per group was included in the in vivo experiments of this study. Although small samples were common practice in early preclinical proof-of-concept phases of CAR-T cell therapy, and we did observe statistically significant differences in the key end points of tumor volume and number of metastases, the reliability of these results should be interpreted with caution [46]. Future confirmatory studies should expand the sample size and include multiple independent experimental batch replicates to enhance the reproducibility of results and provide more reliable evidence support for clinical translation.

This study assessed treatment efficacy over a relatively short observation period; longitudinal follow-up data on long-term persistence of CAR-T cells and potential late toxicity were not examined. This time window may not be sufficient to fully reflect the long-term fate of TROP-2 CAR-T cells in vivo, including their persistence, functional exhaustion dynamics, memory cell formation, and potential late adverse events. In addition, the functional exhaustion of CAR-T cells was also one of the challenges in the treatment of solid tumors. Chronic antigen stimulation and immunosuppressive signals in the tumor microenvironment may lead to the gradual loss of effector function of CAR-T cells [48]. Although we speculated that CBP-mediated remodeling of the tumor microenvironment may partly delay the progression of CAR-T cell exhaustion, this hypothesis still needed to be tested in experiments with long-term observational end points.

Based on the above discussion, more research directions are needed to promote the TROP-2 CAR-T combined with CBP therapy to clinical application: (1) Optimize the animal model and experimental design, and verify the efficacy and safety of the combined therapy in syngeneic mice with sound immune function; The sample size of each group and the observation period are need to be expanded, and multiple independent experimental batches need to be included to enhance the statistical power and reproducibility. Patient-derived xenograft models and vascularized organoid platforms could be used to better simulate the complexity and heterogeneity of human tumor microenvironment [49]. (2) Systematic safety evaluation and optimization of CBP drug structure. A comprehensive bone marrow and hematological safety evaluation system need to be established, the structure of nanoparticles need to be modified to enhance tumor vascular targeting and optimize the dosing regimen, and the best timing combination of CBP and CAR-T cell infusion need to be systematically evaluated. (3) Safety upgrading of engineered CAR-T cells to reduce the off-target risk of TROP-2 as an antigen-specific target, such as using gating system to realize combinatorial antigen logical recognition, integrating inducible safety switches, and adjusting the antigen binding affinity of CAR to achieve selective activation [50]. (4) Explore dual-target or multi-target CAR-T cell design to cope with antigen escape and tumor heterogeneity. (5) Conduct to head-to-head comparison with approved TROP-2 targeted ADCs to explore the triple-combination regimen of TROP-2 CAR-T cells and immune checkpoint inhibitors to maximize the potential of anti-tumor immune response [51].

## Conclusion

TROP-2 has emerged as a promising therapeutic target for breast cancer. Our study indicated that the combination of CBP and TROP-2 CAR-T therapy effectively inhibited both the proliferation and metastasis of breast tumors in vitro and in vivo, thereby providing supportive evidence for advancements in cell therapy for solid tumors.

## Data sharing statement

The dataset supporting the conclusions of this article is included within the article and its additional file. For data that cannot be publicly shared owing to privacy or ethical constraints, requests to access these datasets should be directed to the corresponding author.

## Disclosure

No potential competing interests were reported by the author.

## Ethic approval and consent to participate

The Animal Laboratory of Laboratory animal center Academy of Military Sciences (IACUC-DWZX-2023– 503) approved research involving animals, their data, and biological materials. The Ethics Committee of Chinese PLA General Hospital approved research involving humans, their data, and biological materials (No. S2024– 675– 02). Informed consent was obtained from all human participants prior to sample collection.

## CRediT authorship contribution statement

**Yizhu Chen:** Writing – original draft, Data curation. **LiSheng Wang:** Methodology. **Jian Jiang:** Conceptualization. **YuFan Wei:** Writing – original draft, Formal analysis. **Peng Jiao:** Funding acquisition. **JiaHu:** Methodology. **WeiYuan Zhang:** Methodology. **Jingjin Zhu:** Investigation. **YiMing Wang:** Methodology. **Xiru Li:** Project administration, Conceptualization. **Fengjun Xiao:** Resources, Conceptualization. **Li Zhu:** Funding acquisition, Conceptualization.

## Declaration of competing interest

The authors declare the following financial interests/personal relationships which may be considered as potential competing interests: Li Zhu reports equipment, drugs, or supplies was provided by Changchun Institute of Applied Chemistry, Chinese Academy of Sciences. If there are other authors, they declare that they have no known competing financial interests or personal relationships that could have appeared to influence the work reported in this paper.

## Appendix Supplementary materials

Image, application 1

Image, application 2

Image, application 3
