# Supplementary material for: Combined treatment of TROP‑2 targeted CAR-T and vascular disruptor CBP enhances anti‑tumor activity in triple‑negative breast cancer
Source: Transl Oncol. 2026 May 29;70:102828. doi: 10.1016/j.tranon.2026.102828 (PMC13242033; doi:10.1016/j.tranon.2026.102828)
Supplement: Supplementary file 1 [file mmc1.docx]

**TROP-2 CAR-T Cells Combined with PLG. CA4 Vascular Blocker in Breast Cancer Therapy**

Supplementary file_1. Flow cytometry detection of virus titers

**
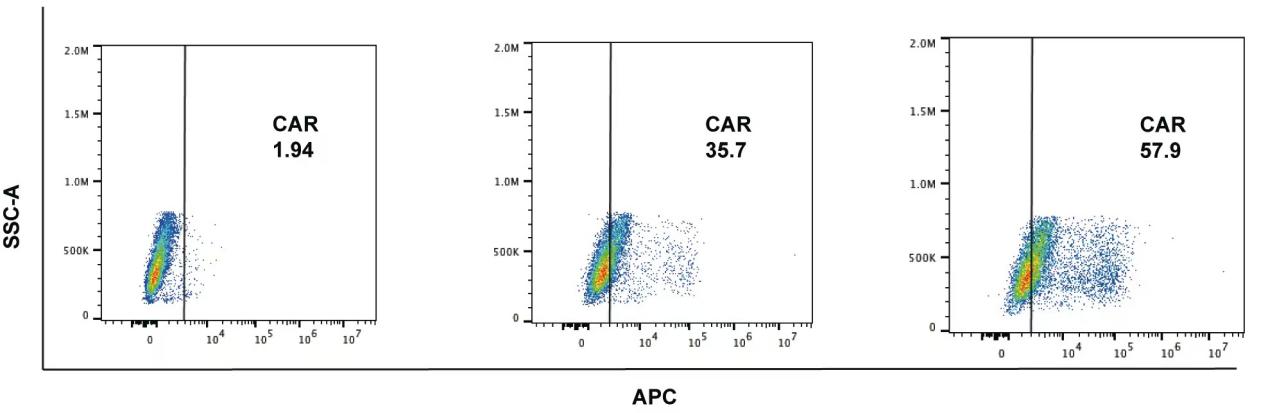
**

Figure S1. Flow cytometry detection of virus titers.
