# Supplementary material for: Combined treatment of TROP‑2 targeted CAR-T and vascular disruptor CBP enhances anti‑tumor activity in triple‑negative breast cancer
Source: Transl Oncol. 2026 May 29;70:102828. doi: 10.1016/j.tranon.2026.102828 (PMC13242033; doi:10.1016/j.tranon.2026.102828)
Supplement: Supplementary file 2 [file mmc2.docx]

**TROP-2 CAR-T Cells Combined with PLG. CA4 Vascular Blocker in Breast Cancer Therapy**

Supplementary file_2. Flow cytometry detection of peripheral blood *in vivo*.

**
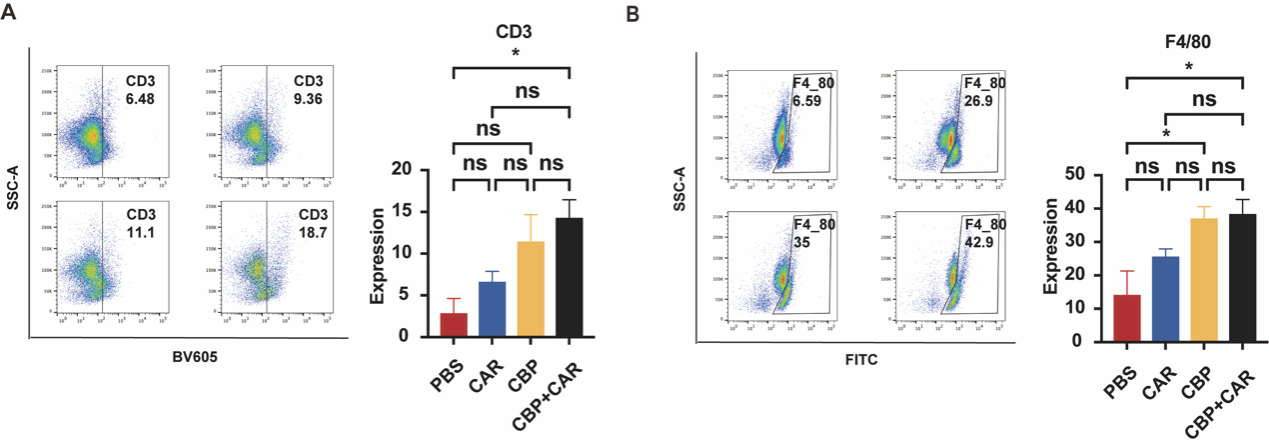
**

Figure.S2. Flow cytometry detection of peripheral blood *in vivo*. A. Expression of CD3+cells of mice in each group (*, *p*<0.05). B. The expression of macrophage cells in each group of mice (*, *p*<0.05).
