# Supplementary material for: Combined treatment of TROP‑2 targeted CAR-T and vascular disruptor CBP enhances anti‑tumor activity in triple‑negative breast cancer
Source: Transl Oncol. 2026 May 29;70:102828. doi: 10.1016/j.tranon.2026.102828 (PMC13242033; doi:10.1016/j.tranon.2026.102828)
Supplement: Supplementary file 3 [file mmc3.docx]

**TROP-2 CAR-T Cells Combined with PLG. CA4 Vascular Blocker in Breast Cancer Therapy**

Supplementary file_3. Additional methods and materials.

Table 1: Experimental Grouping

| Grouping (n=5) | Medication |
| --- | --- |
| PBS group | 200ul PBS |
| CAR-T group | 5x106 cells/200ul |
| CBP group | 20mg/kg body weight, 200ul injection solution |
| CAR-T+CBP group | - |

Table 2: Experimental Processing

| Date | operation |
| --- | --- |
| Day 0 | Grouping, tumor volume measurement |
| Day 2 | After treatment with CBP (20mg/kg body weight, 200ul injection), CAR-T cell therapy (5x106/0ul/mouse) was performed, and mouse body weight and tumor size were simultaneously measured |
| Day 5 | Collect blood and perform flow cytometry to measure the weight and tumor size of mice |
| Day 9 | After treatment with CBP (20mg/kg body weight, 200ul injection), CAR-T cell therapy (5x106/0ul/mouse) was performed, and mouse body weight and tumor size were simultaneously measured |
| Day 12 | Collect blood and perform flow cytometry to measure the weight and tumor size of mice |
| Day 15 | Measure the weight and tumor size of mice |
| Day 19 | Measure the weight and tumor size of mice |
| Day 23 | Measure the weight of mice, euthanize them, collect blood, perform flow cytometry and blood count tests, and extract serum for future use. Take various organs and tumor tissues for HE staining for subsequent pathological detection, extract RNA and WB from mouse tumor tissues for future use. |

Table 3: qPCR Primer List

| Name | Primer Sequence |
| --- | --- |
| H-GAPDH-F | GAAGGTGAAGGTCGGAGTC |
| H-GAPDH-R | GAAGATGGTGATGGGATTTC |
| H-Trop-2-F | ACAACGATGGCCTCTACGAC |
| H-Trop-2-R | GTCCAGGTCTGAGTGGTTGAA |
| WP-primer-F | GGCACTGACAATTCCGTGGT |
| WP-primer-R | AGGGACGTAGCAGAAGGACG |
| M-TNF-α-F | CCCTCACACTCAGATCATCTTCT |
| M-TNF-α-R | GCTACGACGTGGGCTACAG |
| M-IFN-γ-F | ATGAACGCTACACACTGCATC |
| M--IFN-γ-R | CCATCCTTTTGCCAGTTCCTC |
| M-Caspase3-F | AATTCAAGGGACGGGTCATG |
| M-Caspase3-R | GCTTGTGCGCGTACAGTTTC |
